# Ultra‐low LOD H_2_O_2_ Sensor Based on Synergistic Nernst Potential Effect

**DOI:** 10.1002/advs.202413898

**Published:** 2025-04-17

**Authors:** Zhaoqun Wang, Wen Gao, Xiaorong Niu, Yuhang Liu, Zichen Jin, Fan Zhang, Zhengdong Cheng, Xiaoning Jiang, Wendong Zhang, Ting Wang, Jianlong Ji, Xiaojie Chai, Shengbo Sang

**Affiliations:** ^1^ College of Integrated Circuits Taiyuan University of Technology Taiyuan 030024 China; ^2^ Shanxi Key Laboratory of Artificial Intelligence & Micro Nano Sensors Taiyuan 030024 China; ^3^ Xinzhou Comprehensive Inspection and Testing Center Xinzhou 034000 China; ^4^ School of Aeronautics and Astronautics University of Electronic Science and Technology of China Chengdu 611731 China; ^5^ College of Chemical and Biological Engineering Zhejiang University Hangzhou 310058 China; ^6^ Department of Mechanical and Aerospace Engineering North Carolina State University Raleigh 27695 USA; ^7^ State Key Laboratory of Organic Electronics and Information Displays and Jiangsu Key Laboratory for Biosensors Institute of Advanced Materials (IAM) Nanjing University of Posts and Telecommunications Nanjing 210023 China

**Keywords:** hydrogen peroxide detection, organic electrochemical transistors, ultralow limit of detection

## Abstract

The food processing industry and biomedical science research are relying on the low limit of detection (LOD) for hydrogen peroxide (H_2_O_2_). Organic electrochemical transistors (OECTs) are excellent for biochemical sensing applications due to their excellent signal amplification capability. The paper describes  a way of detecting H_2_O_2_ through the use of stacked poly(3,4‐ethylenedioxythiophene): bromothymol blue (PEDOT: BTB)/poly(3,4‐ethylenedioxythiophene): polystyrene sulfonate (PEDOT: PSS) as the semiconducting channel of the OECT. The H_2_O_2_ sensor presents an ultra‐low LOD, down to 1.8 × 10^−12^ M, due to the synergistic effect of the Nernst potential generated by the platinum gate electrode catalyzing H_2_O_2_ and the Nernst potential generated by the interaction between BTB molecules and hydrogen ions, the by‐product of H_2_O_2_ catalysis. A microsystemwith a signal processing circuit and a mobile app for the sensor has been developed, and they are then tested on commercial milk samples to verify their reliability. Since the majority of enzyme‐catalyzed reactions generate or use H_2_O_2_ in biochemical reactions, the methodology is applicable not only to the detection of H_2_O_2_ but also to the detection of analytes based on enzyme‐catalyzed reactions. For demonstration, glucose detection with a LOD of down to 8.82 × 10^−11^ M is also presented.

## Graphical abstract

Due to the synergistic effect of $E_{\mathrm{Nernst},\;H_{2}O_{2}}$ and $E_{\mathrm{Nernst},\;H^{+}}$, the proposed sensors in this study can achieve ultralow H_2_O_2_ detection limit as low as 1.8 × 10^−12^ M based on the stacked PEDOT: BTB/PEDOT: PSS semiconducting layer. Furthermore, this innovative approach to reducing LOD is also broadly applicable to analytes detected via enzyme‐catalyzed reactions.

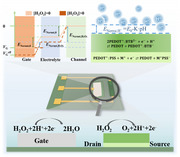

## 1 Introduction

Hydrogen peroxide (H_2_O_2_) is a vital analyte because it plays a role in multiple physiological and pathological processes.^[^ ^1^, ^2^, ^3^ ^]^ Specifically, H_2_O_2_ is the by‐product of several enzyme‐catalyzed biochemical reactions involving lactate oxidase, glutamate oxidase, and glucose oxidase.^[^ ^4^ ^]^ Detecting H₂O₂ with a low detection limit is crucial for studying these enzyme activities, especially in metabolic pathways. In addition, H₂O₂ is used as a disinfectant in food packaging and as a bleaching agent. Detecting residual H₂O₂, which must be effectively controlled within permissible limits,^[^ ^5^ ^]^ is essential for ensuring food and beverage safety, as excessive levels can harm consumers. There are various H_2_O_2_ detection methods, such as spectroscopy, chromatography, and chemiluminescence.^[^ ^6^, ^7^ ^]^ However, these detection devices are usually bulky and expensive, and the detection methods are often complex, requiring trained personnel to operate, dramatically limiting their widespread applications, especially in on‐site or mobile detections.

Organic electrochemical transistors (OECTs) are highly suitable for portable biochemical sensing applications due to no need for bulky and non‐polarizable electrodes and their low operating voltage, excellent flexibility, and biocompatibility characteristics.^[^ ^8^, ^9^ ^]^ Fabio employed platinum (Pt) as the source, drain, and gate material, while poly(3,4‐ethylenedioxythiophene): polystyrene sulfonate (PEDOT: PSS) as the semiconducting material to construct OECTs‐based H_2_O_2_ sensors.^[^ ^10^ ^]^ The linear detection region is 5–10^3^ µm, and the limit of detection (LOD) is 5 µm. Xiang et al. employed a screen‐printed carbon paste electrode modified with carbon nanotubes and Pt nanoparticles as the gate electrode and used PEDOT: PSS film as the active layer to construct an OECT‐based H_2_O_2_ sensor,^[^ ^11^ ^]^ which exhibits a linear region of 0.5 to 100 µm and a 0.2 µm LOD of H_2_O_2_. Guangyu et al. presented donor‐acceptor ambipolar polymer‐based OECTs, which can offer real‐time and highly sensitive H_2_O_2_ detection in a linear region of 1 nm to 100 µm and a 1 nm LOD.^[^ ^12^ ^]^ All these sensors perform low LOD detection mainly because the Nernst potential generated by the Pt gate electrode‐catalyzing H_2_O_2_ could regulate the electrochemical doping state of the semiconducting layer, thereby utilizing the excellent signal amplification capability.

BTB (bromothymol blue) is one of the most used chemical indicators for weak acids and bases.^[^ ^13^ ^]^ Previous studies show that both OECTs based on the PEDOT: BTB modified gate electrode and OECTs based on the PEDOT: BTB/PEDOT: PSS stacked semiconducting layer could realize hydrogen ion sensing due to the interaction between hydrogen ions and BTB molecules.^[^ ^14^, ^15^ ^]^ Considering hydrogen ions are the by‐product of H_2_O_2_ catalytic reactions, we speculate that introducing PEDOT: BTB into the traditional OECT‐based H_2_O_2_ sensor is beneficial for more efficient utilization of the electrochemical potential variations generated by the cascade reaction, thus providing a new approach for H_2_O_2_ sensor optimizations.

**Scheme 1:**
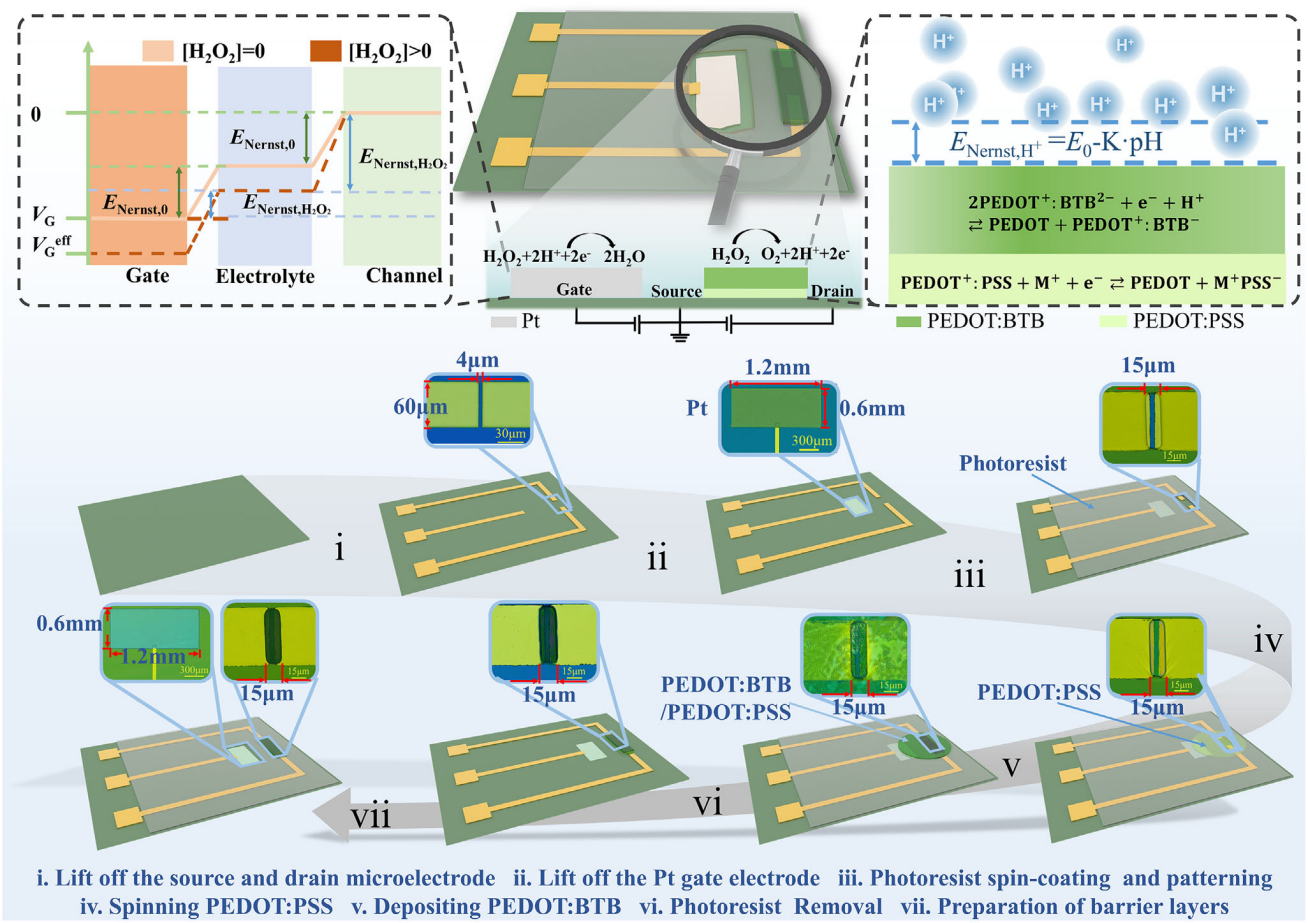
The diagram of the operating mechanism and the fabrication method of the H_2_O_2_ sensor.

The current work demonstrates that constructing OECTs based on the stacked PEDOT: BTB/PEDOT: PSS layer can significantly improve the H_2_O_2_ sensors’ detection limit due to the synergistic Nernst potential effect. As shown in **Scheme** **1**, the Pt electrode catalyzes the H_2_O_2_ molecules, leading to a Nernst potential and changing the semiconducting layer's free carrier concentration by ion electrochemical doping. At the same time, the reaction between hydrogen ions and the BTB molecules brings in another Nernst potential that could directly change the electrical potential of the buried PEDOT: PSS layer. As such, the two Nernst potentials mentioned above can synergistically act on the semiconducting layer, thereby endowing the sensor with better performance. As a demonstration, the micro‐nano manufacturing approach fabricates the stacked semiconducting layer with a footprint of only 4 microns and a thickness of 361 nm, and we present a microsystem consisting of a signal processing circuit and a mobile app, which achieved an H_2_O_2_ detection limit as low as pM and could trace H_2_O_2_ molecules in milk samples. Based on the H_2_O_2_ detection methodology, a glucose sensor with LOD down to 8.82 × 10^−11 ^M is also presented.

## 2 Results and Discussion

### 2.1 The Stacked Layer and its Photoelectrochemical Characteristics

**Figure 1:**
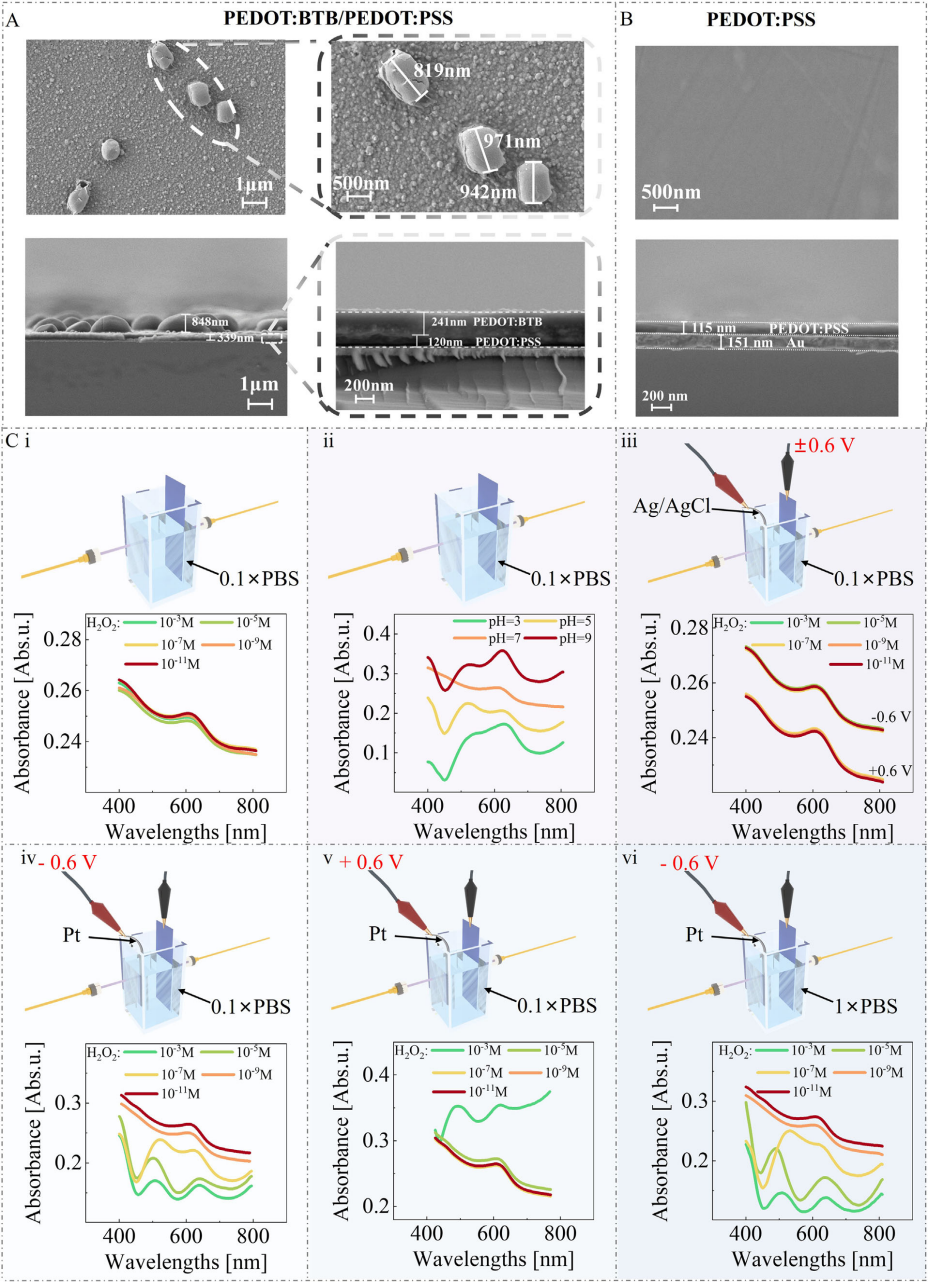
Morphology and photoelectrochemical characterizations of single PEDOT: PSS layer and stacked PEDOT: BTB/PEDOT: PSS layer. A) The SEM image of stacked PEDOT: BTB/PEDOT: PSS layer. B) The SEM image of a single PEDOT: PSS layer. C) The UV–vis characterizations are conducted by i) immersing the stacked layer in 0.1 × PBS mixed with different concentrations of H_2_O_2_; ii) immersing the stacked layer in 0.1 × PBS with varying conditions of pH; iii) immersing the stacked layer in 0.1 × PBS mixed with different concentrations of H_2_O_2_ and biasing the stacked layer with ±0.6 V versus Ag/AgCl electrode. The UV–vis characterizations are conducted by immersing the stacked layer in 0.1 × PBS mixed with different concentrations of H_2_O_2_ and biasing the Pt electrode with iv) −0.6 V versus the stacked layer, or v) +0.6 V versus the stacked layer; (vi) immersing the stacked layer in 1 × PBS and biasing the Pt electrode with −0.6 V versus the stacked layer.

Firstly, the morphology of the stack layer and the electrochemical/photoelectrochemical properties of the electrolyte/stack layer interface have been investigated. As shown in **Figure** **1**A,B, the single layer has a relatively smooth surface. Still, it gets much rougher after PEDOT: BTB electrodeposition (Note S3, Supporting Information), and many granular structures can be observed on the PEDOT: BTB films. From the cross‐sectional view of the SEM image (Figure 1A,B), the thickness of the spin‐coated PEDOT: PSS film and electrodeposited PEDOT: BTB film is found to be 120 and 241 nm, respectively.

The photoelectrochemical investigations are first conducted in 0.1 × PBS. Figure 1C‐i,ii illustrate that the H_2_O_2_ concentration does not affect the absorption spectra of the semiconducting layer, but the concentration of hydrogen ions does. These results could confirm the electrochemical reaction between PEDOT: BTB and hydrogen ions previously proposed by Federica and our group:^[^ ^14^, ^15^, ^19^, ^20^ ^]^

(1) $$2\mathrm{PEDO}T^{+}:\mathrm{BT}B^{2-}+e^{-}+H^{+}⇄\mathrm{PEDOT}+\mathrm{PEDO}T^{+}:\mathrm{BT}B^{-},$$

Which could further induce a Nernst potential expressed as:

(2) $$E=E^{0}+\frac{\mathrm{RT}}{F}\cdot \ln\frac{\left[2\mathrm{PEDO}T^{+}:\mathrm{BT}B^{2-}\right]\cdot \left[H^{+}\right]}{\left[\mathrm{PEDOT}\right]\cdot \left[\mathrm{PEDO}T^{+}:\mathrm{BT}B^{-}\right]}.$$

*E* ^0^ is the standard redox potential of PEDOT^+^/PEDOT, *E* is the electrochemical potential of PEDOT: BTB thin films, *R* is the gas constant, *T* is the temperature, and *F* is the Faraday constant. Even if the stacked layer is biased with a voltage, the UV–vis absorption spectra obtained at different H_2_O_2_ concentrations still overlap (Figure 1C‐iii).

Then, a two‐electrode electrochemical system consisting of Pt and stacked layer‐modified indium tin oxide electrodes (Figure 1C‐iv,v) is constructed, where the absorption spectrum changes with the H_2_O_2_ concentration. As such, we speculate that the catalytic reaction of H_2_O_2_ (Equations 3 and 4) will alter the local H^+^ concentration on the surface of the stacked semiconducting layers and thus lead to the variation of the Nernst potential (Equation 2), which tunes the electrochemical state of the stacked layer and the current flowing source‐drain loop (*I*_DS_) in consequence.^[^ ^21^, ^22^, ^23^ ^]^

(3) $$H_{2}O_{2}⇋O_{2}+2H^{+}+2e^{-}\;\;\;\mathrm{at}\;\mathrm{relative}\;\mathrm{positive}\;\mathrm{bias},$$

and

(4) $$H_{2}O_{2}+2H^{+}+2e^{-}⇋H_{2}O\;\;\;\mathrm{at}\;\mathrm{relative}\;\mathrm{negative}\;\mathrm{bias}.$$

Moreover, by comparing Figure 1C‐iv,v, it can be found that when the Pt electrode bias is −0.6 V, the absorption spectrum can be better distinguished at H_2_O_2_ concentration as low as 10^−11^ M, providing the experimental evidence for the effect of Nernst potential on the channel has contributed significantly to optimizing the device's LOD.

It should be noted that to eliminate the influence of conductivity on the experimental results, the above experiments and the subsequent OECT performance characterization are all conducted in 0.1 × PBS with a conductivity of 34.39 ± 0.73 mS⋅cm^−1^. We also investigate the UV–vis absorption spectra of the stacked layer in 1 × PBS and 0.1 M NaCl (Figure 1C‐vi; Note S4, Supporting Information). Notably, it is evident that the absorption spectrum exhibits no significant alteration compared to 0.1 × PBS. This information suggests that the kinetic process of the electrochemical reaction between PEDOT: BTB and H^+^ is faster than that of PBS buffering. Even if the buffering effect may influence H^+^ concentration variations, the locally high concentration of hydrogen ions can still regulate the electrochemical state of the stacked semiconducting layer.

### 2.2 The Synergistic Nernst Potential Effect and the Operating Point Setting Method

**Figure 2:**
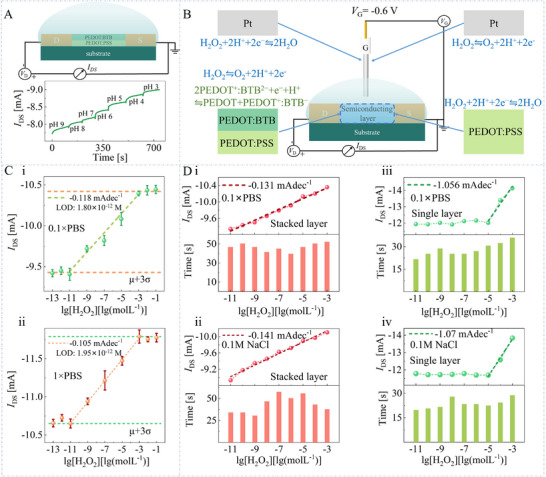
The operating mechanism of stacked semiconducting layer‐based H_2_O_2_ sensor. A) The *I*_DS_ is recorded when the stacked semiconducting channel is exposed to electrolytes with different pH. B) The mechanism diagram of OECTs using the stacked PEDOT: BTB/PEDOT: PSS film and single PEDOT: PSS film as the semiconducting layer, respectively. C) Comparing the device performance when using 1 × PBS and 0.1 × PBS as the electrolyte for the OECT with stacked semiconducting layer. D) Comparing the device performance when using 0.1 × PBS and 0.1 m NaCl as the electrolyte for the OECT with stacked PEDOT: BTB/PEDOT: PSS and single PEDOT: PSS semiconducting layer.

Based on the stacked PEDOT: BTB/PEDOT: PSS layer, we first construct a two‐terminal OECT gated by the pH solution. As shown in **Figure** **2**A, the absolute value of the *I*_DS_ flowing in the source‐drain loop increases as the pH decreases, indicating that the electrochemical state of the stacked semiconducting layer could be tuned by the Nernst potential (Equation 2) induced by the electrochemical reaction between PEDOT: BTB and hydrogen ions (Equation 1). Next, we construct an OECT‐based H_2_O_2_ sensor by introducing a Pt electrode as the OECT's gate electrode (Figure 2B). At a gate bias of −0.6 V, the sensor obtains a wider linear range (10^−11^–10^−3^ M) than the device constructed by the single PEDOT: PSS semiconducting layer. In particular, Figure 2C‐i illustrates that the detection limit can be down to 1.8 × 10^−12^ M in 0.1 × PBS, which is four orders of magnitude lower than traditional OECT‐based H_2_O_2_ sensors (Note S5, Supporting Information).

The mechanism for the ultralow limit detection of H_2_O_2_ could be elucidated as the synergistic Nernst potential effect (Figure 2B). Firstly, the Nernst potential generated by the H_2_O_2_ catalytic reaction (Equations 3 and 4) could be expressed as:

(5) $$E_{\mathrm{Nernst},\;H_{2}O_{2}}=E^{0}\;-\frac{\mathrm{kT}}{2e}\ln\left(\left[H_{2}O_{2}\right]\right)\;\mathrm{at}\;\mathrm{the}\;\mathrm{channel},$$

and

(6) $$E_{\mathrm{Nernst},\;H_{2}O_{2}}=E^{0}+\frac{\mathrm{kT}}{2e}\ln\left(\left[H_{2}O_{2}\right]\right)\;\mathrm{at}\;\;\mathrm{the}\;\;\mathrm{gate}.$$

When the H_2_O_2_ concentration increases, the electrolyte's potential decreases. As the source and drain bias fix the electric potential of the semiconducting layer, the reduced electric‐potential difference drives ions to leave the PEDOT: PSS semiconducting layer. Consequently, the hoes dope the semiconducting layer, gradually increasing the |*I*_DS_|. Secondly, hydrogen ions generated near the channel (Equation 2) will interact with the PEDOT: BTB layer, generating another Nernst potential:

(7) $$E_{\mathrm{Nernst},\;H^{+}}=E^{0}+\frac{\mathrm{kT}}{e}\cdot \ln\left[H^{+}\right],$$

or

(8) $$E_{\mathrm{Nernst},\;H^{+}}=E^{0}\;-\frac{\mathrm{kT}}{e}\ln10\cdot \mathrm{pH}.$$

Due to the direct contact between PEDOT: BTB and PEDOT: PSS, the increased electrical potential of the PEDOT: BTB can directly oxidize the buried PEDOT: PSS film. Thus, as the H_2_O_2_ concentration increases, the H^+^ increases, leading to the increment of |*I*_DS_| (Figure 2C,D).

We compare the sensing performance of the Pt‐gate OECTs with different channels at *V*_G_ = −0.6 V in Figure 2D. As shown in Figure 2D‐i, once H_2_O_2_ electrocatalysis produces the first Nernst potential of $E_{\mathrm{Nernst},\;H_{2}O_{2}}$, the accompanying hydrogen ions will further react with PEDOT: BTB to produce $E_{\mathrm{Nernst},\;H^{+}}$. These two Nernst potentials work together, producing a synergistic signal amplification effect. The production of hydrogen ions during catalytic reactions can be quickly reacted with PEDOT: BTB, resulting in the detection of hydrogen peroxide even at low H_2_O_2_ concentrations. However, for the OECT constructed by the single PEDOT: PSS semiconducting layer (Figure 2D‐iii), due to the lack of interaction between hydrogen ions and PEDOT: BTB, the synergistic effect no longer exists, leading to the LOD being far inferior to the stacked layer device. To eliminate the influence of PBS buffer on the local changes in hydrogen ion concentration, we measure the sensor response by spiking different concentrations of H_2_O_2_ in 1 × PBS (Figure 2C‐ii) and 0.1 M NaCl (Figure 2D‐iii,iv). It is found that neither the sensitivity, the linear range, nor the LOD of the sensor has been significantly affected, consistent with the results of the photoelectrochemical investigations.

**Figure 3:**
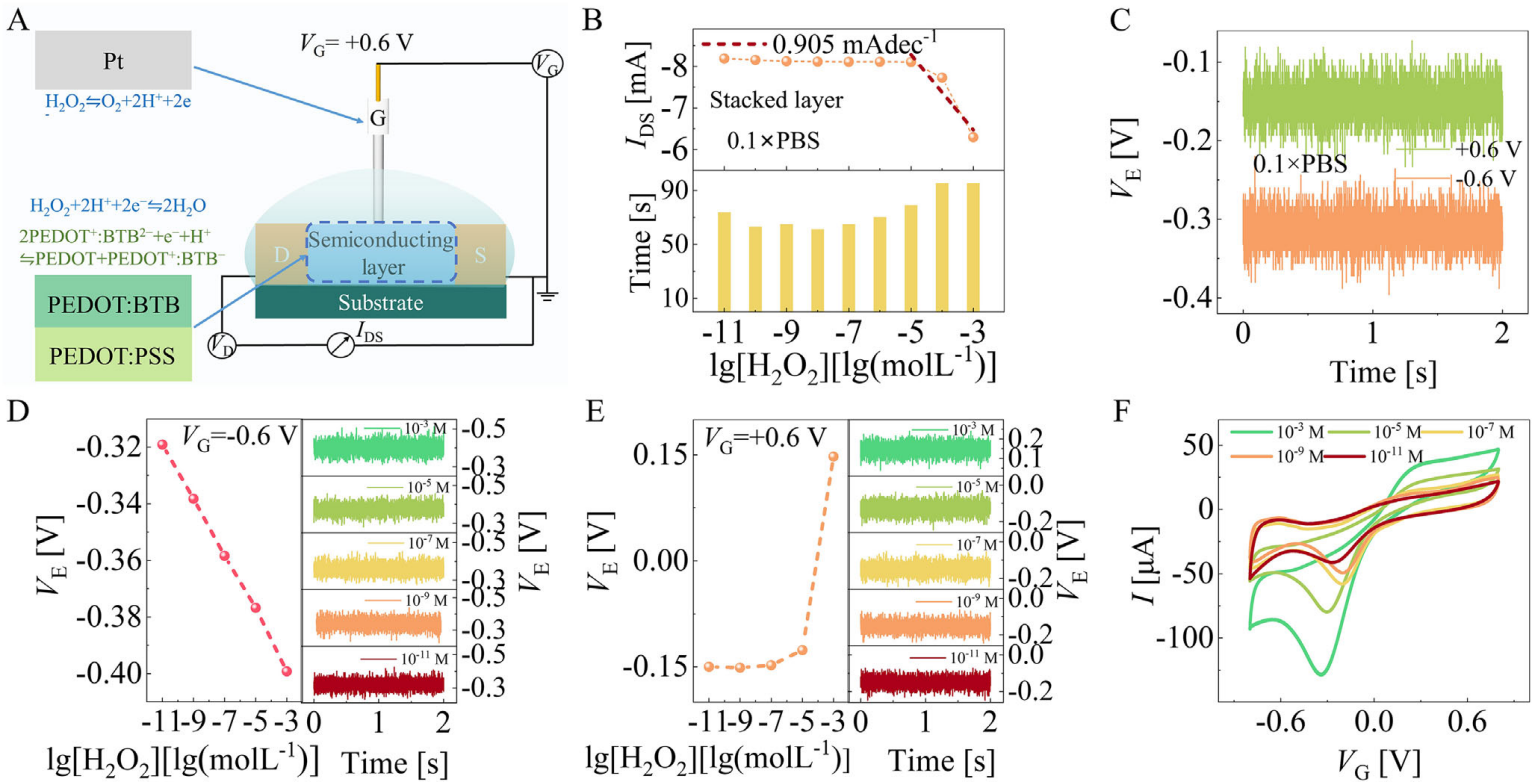
The mechanism of the gate bias (*V*_G_) operating point affects the sensor performance. A) The mechanism diagram of OECTs with the stacked PEDOT: BTB/PEDOT: PSS film as the semiconducting layer under a +0.6 V gate bias. B) Sensor performance is obtained when stacked semiconducting layer‐based OECT is operated under a *V*_G_ of +0.6 V. C) The *V*_E_ is measured in 0.1 × PBS without mixing H_2_O_2_. The *V*_E_ is obtained in 0.1 × PBS with different concentrations of H_2_O_2_ under a *V*_G_ of D) −0.6 V and E) +0.6 V, respectively. F) CVs of different concentrations of H_2_O_2_ catalyzed by Pt electrode in the three‐electrode electrochemical system.

In addition to the experimental setup of the stacked semiconducting layer and Pt gate electrode, the gate bias (*V*_G_) operating point setting is also crucial for sensor performance optimizations. When the gate bias is set as +0.6 V with the transistor structure unchanged, the electrochemical reaction at the gate and the semiconducting layer reverses (**Figure** **3**A). Though the synergistic effect still exists, where $E_{\mathrm{Nernst},\;H_{2}O_{2}}$ increases while *E*_Nernst, H_^+^ decreases with increasing H_2_O_2_ concentration, leading to a decreasing |*I*_DS_| (Figure 3B). However, we find that the LOD seriously deteriorates. To figure out the reason, we measure the electrolyte potentials (*V*_E_) by introducing another Ag/AgCl electrode (Note S6, Supporting Information) into the OECT to determine how the *V*_G_ influences the sensor performance. As shown in Figure 3C, the obtained *V*_E_ is −0.15 V for *V*_G._ = +0.6 V and −0.31 V for *V*_G._ = −0.6 V, respectively. Because the Ag/AgCl electrode and the source electrode are electrically short‐connected, the electrical potential varies between *V*_E_ and *V*_G_, which is +0.75 V for *V*_G._ = +0.6 V and −0.29 V for *V*_G._ = −0.6 V, could be regarded as the Pt electrode's working potential.

Based on the configuration, Figure 3D,E shows the *V*_E_ obtained at different gate biases and under different H_2_O_2_ concentrations. It can be seen that when *V*_G_ is −0.6 V, *V*_E_ shows better linearity but a smaller fluctuation amplitude changing with the H_2_O_2_ concentrations, which endows the sensor operated under *V*_G_ of −0.6 V with a broad linear region while the sensor operated under *V*_G_ of +0.6 V with a high sensitivity (Figures 2D‐i and 3B). The CV investigations could also elucidate why the sensor operated under *V*_G_ of −0.6 V presents an improved LOD. Figure 3F shows the CV results of different concentrations of H_2_O_2_ catalyzed by the Pt electrode investigated in the three‐electrode electrochemical system, from which one can find that the Pt electrode has better catalytic efficiency at a working potential of −0.29 V than +0.75 V.^[^ ^24^, ^25^ ^]^ In particular, the redox current obtained in the CV is −0.041 mA at −0.29 V (corresponding to *V*_G_ = −0.6 V) and +0.020 mA at +0.75 V (corresponding to *V*_G_ = +0.6 V), respectively. It could be found that the electrical potential difference of −0.29 V obtained by *V*_E_ of −0.31 V and *V*_G_ of −0.6 V is close to the reduction potential of −0.275 V under 10^−11^ M concentration of H_2_O_2_. A large redox current means more hydrogen ions are involved in the reaction (Equations 3 and 4), generating more significant Nernst potential variations, making OECT's current output sufficient to be detected even at low H_2_O_2_ concentrations. Therefore, in addition to the synergistic effect of Nernst potential, selecting an appropriate working point of the gate potential is also necessary for achieving an ultralow detection limit for H_2_O_2_ detection.

### 2.3 The Hindering Effect of PEDOT: BTB on Ion Transport

Although the use of stacked semiconducting layers improves the sensor LOD, the resulting design deteriorates the sensitivity and response speed of the device. As shown in Figure 2D‐i,iii, the sensitivities of stacked layer and single layer devices are −0.131 and −1.056 mA·dec^−1^, respectively. The hindering effect of PEDOT: BTB on ion transport may be the leading cause of this phenomenon, which the photoelectrochemical, EIS, and wettability characterizations could verify the mechanism.

**Figure 4:**
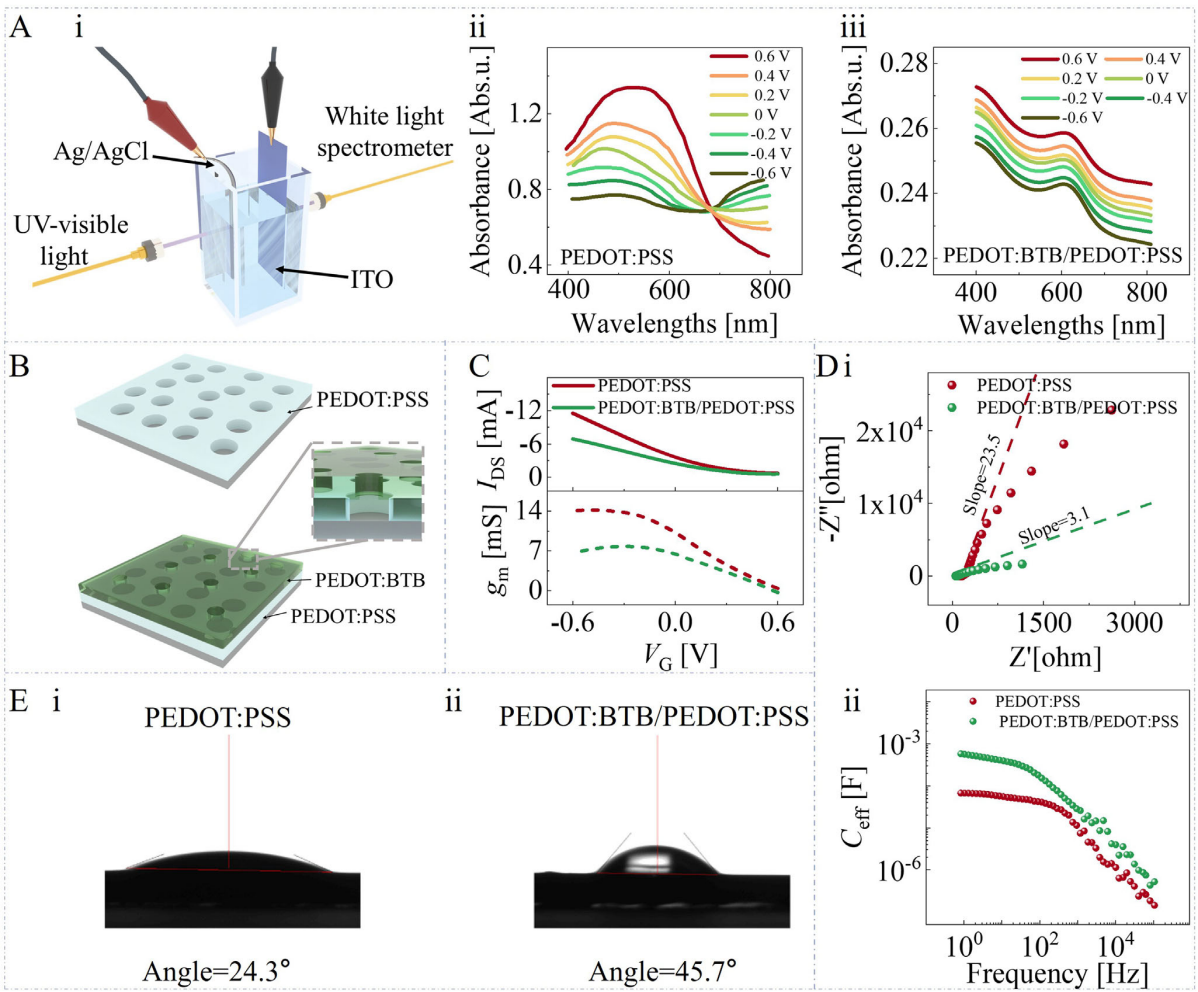
Investigations for elucidating the hindering effect. A) The schematic diagram of the experimental setup and the experimental results of photoelectrochemical investigations. B) Structural diagram of single PEDOT: PSS layer and stacked PEDOT: BTB/PEDOT: PSS. C) The steady‐state characterization is obtained using an Ag/AgCl electrode as the gate electrode. D) EIS characterizations. E) Wettability characterizations.

As shown in **Figure** **4**A, the single layer presents a stronger absorption and a more significant absorption intensity regulation in the bias range of −0.6–+0.6 V in the vicinity of 600 nm compared to the stacked layer. As such, we infer that the PEDOT: BTB layer has smaller and fewer ion transport channels than PEDOT: PSS films (Figure 4B). Due to the blocking effect of the PEDOT: BTB layer on ions, the Ag/AgCl gate electrode has a better tuning ability (indicated by the transconductance peak (*g*_m, max_) of 14.08 mS) on the single layer compared to the stacked layer (6.58 mS) (Figure 4C). The electrochemical impedance investigation results can also verify the blocking effect. As shown in the Nyquist plot of Figure 4D‐i, both the slopes obtained by the stacked layer and the single layer exceed 1 in the low‐frequency region, and the slope of the single layer (23.5) is much larger than the one of the stacked layer (3.1), attributed to the more easily accessible channels for electrolyte ions. Figure 4E shows the wettability test results, where both single PEDOT: PSS layer with a contact angle of 24.3° and stacked PEDOT: BTB/PEDOT: PSS layer with a contact angle of 45.7° present hydrophilic characteristics, which means that wettability does not significantly affect the ion transport process, and thus, it is mainly the reduction of ion channels that causes the hindering effect. We also find that the single‐layer devices have a more significant |*I*_DS_| under the same Ag/AgCl gate bias, which can be verified by the EIS results. As shown in Figure 4D‐ii, the effective capacitance estimated from the Nyquist plot using the out‐of‐phase impedance (Z″ = 1/2π*fC*_eff_) for the stacked layer and the single layer is 561.2 µF and 66.7 µF at a low frequency of 1 Hz, respectively. As such, when modified PEDOT: PSS layer with PEDOT: BTB, the solid/liquid interface capacitance has increased by nearly an order of magnitude, indicating a reduced voltage drop on the semiconducting layer, and hence, the stacked layer devices present a smaller |*I*_DS_|_._

The hindering effect of PEDOT: BTB on ion transport not only impacts the sensor sensitivity (as discussed in Figure 3) but also influences the response speed of OECT and the as‐prepared sensor (Note S7 in the Supporting Information). As shown, the response time of Pt electrode‐gated stacked‐layer devices (Figure S5D‐iii, Supporting Information) to the *V*_G_ variations is generally longer than that of single‐layer devices (Figure S5C‐iii, Supporting Information), like the Ag/AgCl electrode‐gated devices (Figure S5A‐iii,B‐iii, Supporting Information). The hindering effect on the sensor's response speed can also be observed in Figure 2. The response time of sensors to the H_2_O_2_ concentration variations obtained in Figure 2D‐i,iii is averaged and marked as τ_i_, τ_ii_, which presents such a relationship: τ_ii_ (28.01 s) < τ_i_ (46.60 s).

### 2.4 The Ultralow LOD Biosensors Characterizations

**Figure 5:**
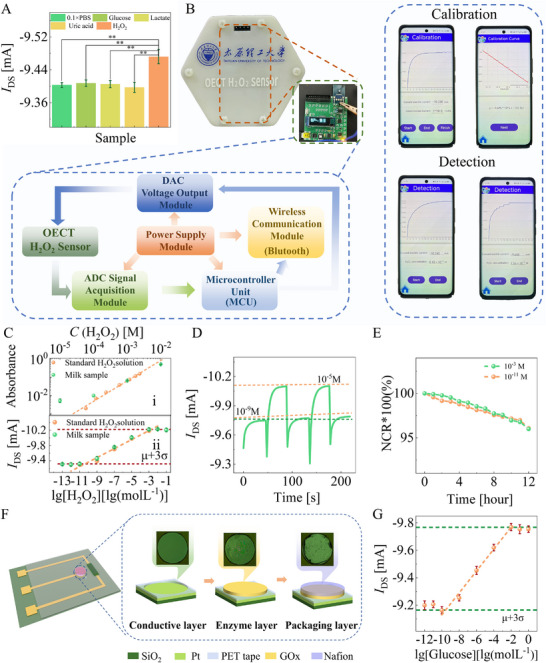
The ultralow LOD biosensor. A) Four tests are applied for selectivity characterizations. The statistical analysis is performed using a one‐way analysis of variance, where ^**^ *p* < 0.01. B) Optical image of interface circuit and mobile app. C) Tracing H_2_O_2_ levels in the complex samples, milk, by i) the national standard method and ii) the OECT‐based biosensor. The error bar indicates the standard error of three independent devices. D) The hysteresis width, and E) the drift rate. F) The fabrication process of the gate electrode of the as‐proposed glucose sensor. G) The glucose sensor calibration. Herein, the error bar indicates the standard error of three independent devices.

H_2_O_2_ is the primary chemical used to sterilize plastic packaging material in the milk industry. Although H_2_O_2_ breaks down naturally, it may still be present in milk. China, the United States, Europe, Japan, and other countries^[^ ^26^ ^]^ have banned H_2_O_2_ residue in milk since the excess concentrations of H_2_O_2_ in milk result in many adverse effects on humans, such as cardiovascular diseases, severe gastrointestinal problems, and neurodegenerative disorders.^[^ ^27^ ^]^ The OECT with a design of a large width/length ratio (15:1) and a short channel design (4 µm) is finally adopted to compensate for the adverse effects. **Figure** **5**A shows the selectivity investigation results of the proposed H_2_O_2_ sensor, where the test is conducted by adding glucose, lactate, and H_2_O_2_ of 10^−11^ M concentration in a 0.1 × PBS in sequence. The maximum change in semiconducting channel current (*I*_DS_) is the only 0.004 ± 0.009 mA for interferences, one order of magnitude lower than the sensor's response to H_2_O_2_ molecules (0.069 ± 0.014 mA). We also develop the peripheral circuits (Note S8, Supporting Information) and upper computer for the proposed H_2_O_2_ sensor (Figure 5B). In brief, five modules are integrated into the peripheral circuit, including the power supply module, the voltage output module, the signal acquisition module, the microcontroller unit (MCU), and the wireless communication module. The collected data is wirelessly transmitted to the upper computer through Bluetooth in real‐time. The upper computer consists of the front and back end, encoded in Java. The back end receives data from the Bluetooth transmission, decodes and processes data, and performs linear regression fitting. The front end is a mobile app developed by the Android Studio that uses WebView to plot the current response and uses the Android X library to implement a human‐machine interaction interface.

The recovery experiments are conducted to explore the feasibility of the proposed ultra‐low LOD H_2_O_2_ sensors in actual samples (Figure 5C). In particular, commercially available milk, instead of PBS alone, is used as an electrolyte sensor. First, we use the national standard method of GB 5009.226‐2016 (the titanium salt colorimetry method published by the National Health and Family Planning Commission of China) (Note S9, Supporting Information) to detect H_2_O_2_ in the milk. The principle of the national standard method is as follows: In an acidic solution, H_2_O_2_ can react with titanium ions to form a stable orange complex. At a wavelength of 430 nm, the absorbance is directly proportional to the content of H_2_O_2_ in the sample, and using the colorimetric method can determine the content of H_2_O_2_ in the sample. Considering that no H_2_O_2_ could be found in such a sample, we spike H_2_O_2_ in milk and conduct recovery experiments thereafter. As shown in Figure 5C‐i, the national standard method can only detect H_2_O_2_ within the linear range of 10^−2^–10^−4^ M with a LOD of 8.8 × 10^−5^ M. Moreover, only the high concentrations of H_2_O_2_ between 10^−2^ and 10^−4^ M present satisfying recovery (Table S2, Supporting Information). By comparison, our proposed H_2_O_2_ sensor presents a gratifying recovery range from 93.47% to 102.24%, with an excellent relative standard deviation of 2.87% – 7.23% when detecting H_2_O_2_ in much lower concentration (Figure 5C‐ii, Table S3, Supporting Information). The obtained LOD of H_2_O_2_ in milk is 5.02 × 10^−11^ M, and the sensitivity is −1.01 × 10^−4^ A·dec^−1^.

The stability and reliability of the proposed sensors are evaluated by measuring the hysteresis width and drift rate. The hysteresis width is defined as the *I*_DS_ difference between the initial and final electrolyte solution at the same H_2_O_2_ level; the drift rate is determined as a variation of the NCR = $\Delta I_{DS}/I_{DS,0}$ per hour. Figure 5D illustrates that the hysteresis width in the 10^−9^ and 10^−5^ M loops is 0.041 mA, about 0.42% of the initial *I*_DS_. Moreover, the maximum drift rate obtained is 0.23%·h^−1^ and 0.28%·h^−1^ at 10^−9^ and 10^−5^ M, respectively (Figure 5E). All these results suggest that the proposed sensor and measurement method holds broad application prospects in H_2_O_2_ sensing in the complex matrix.

We also achieve ultra‐low LOD glucose detection based on the synergistic Nernst potential effect. As shown in Figure 5F, the glucose sensor is fabricated by employing the Nafion‐GOx‐Pt stacked layer as the gate electrode and using the PEDOT: BTB/PEDOT: PSS stacked layer as the semiconducting layer of the OECTs (Experimental Section‐Fabrication of OECTs‐Based Ultralow LOD Glucose Sensor). Under a gate bias of −0.6 V, the enzymatic reaction involved with the added glucose is catalyzed by GOx, producing H_2_O_2_ and D‐glucono‐1,5‐lactone (Equation 9).^[^ ^16^ ^]^ Subsequently, H_2_O_2_ is oxidized at the semiconducting channel, yielding hydrogen ions (Equation 3):

(9) $$D-\mathrm{Glucose}\overset{\mathrm{Enzyme}\left(\mathrm{GOx}\right)}{\to }D-\mathrm{glucono}-1,5-\mathrm{lactone}+H_{2}O_{2},$$

and inducing the electrochemical reaction between hydrogen ions with PEDOT: BTB (Equation 1).

Finally, a LOD of glucose down to 8.82 × 10^−11 ^M could be realized, which can be compared with the state‐of‐the‐art glucose sensors (Note S10, Supporting Information). Considering that H_2_O_2_ is a by‐product of many enzyme‐catalyzed reactions, the structure is expected to be widely used in enzyme sensors in the future.

## 3 Conclusion

We have achieved ultralow LOD H_2_O_2_ detection based on stacked PEDOT: BTB/PEDOT: PSS layers. Due to the synergistic effect of $E_{\mathrm{Nernst},\;H_{2}O_{2}}$ and $E_{\mathrm{Nernst},\;H^{+}}$, the proposed sensors can detect H_2_O_2_ concentrations as low as 1.8 × 10^−12^ M, which is the lowest LOD reported so far. Moreover, it is a brand‐new methodology for reducing LOD, which not only applies to H_2_O_2_ detection but also can be widely used for analyte detection based on enzyme‐catalyzed reactions.

## 4 Experimental Section

Detailed information about the reagents, instruments, microelectrodes chip fabrication process, charge‐transport‐layer preparation process, and solution sample configuration method are attached in Note S1 (Supporting Information).

### Fabrication of the Stacked Sensing Layers

For the preparation of the stacked layers, a mixed aqueous solution consisting of 10 mm 3,4‐ethylenedioxythiophene (EDOT), 1 mm BTB, 1 mm phosphate buffer solution (PBS), and 0.1 m KNO_3_ was deposited by the cyclic voltammetry (CV) method on the spin‐coated PEDOT: PSS film. In detail, the preset source (S) and drain (D) electrodes connected by spin‐coated PEDOT: PSS film served as the working electrode; the saturated calomel electrode served as the reference electrode; the Pt wire served as the counter electrode. An electric potential ranging from 0 to 1 V was then applied to the working electrode at a scan rate of 0.1 V s^−1^ for 15 cycles (Note S2, Supporting Information). Finally, the electrodeposits were cleaned with deionized water and dried at room temperature.

### Fabrication of OECTs‐Based H_2_O_2_ Sensor

We first prepared microelectrodes through traditional microfabrication to construct the H_2_O_2_ sensor. In detail, AZ 400K developer was used to pattern the photoresist ROL‐7133 on a silicon substrate with a silicon oxide layer by photolithography (FC‐25S, Chengdou Hengcheng Photoelectric Technology Co., Ltd, China). Then, 20‐nanometer titanium and 100‐nanometer gold thin films were deposited using dual‐target magnetron sputter equipment (VTC‐600‐2HD, Shenyang Kejing Automation Equipment Co., Ltd, China). The film was used as the adhesive layer between Au and SiO_2_ films. The source and drain microelectrode were fabricated using the lift‐off process, where the distance between the source and drain determined the length of the semiconducting channel.

Based on the second lift‐off process, a Pt gate electrode with a thickness of 100 nm was deposited on the SiO_2_ substrate (Scheme 1). The third lift‐off process opened the window for patterning the semiconducting channel. Then, the PEDOT: PSS film was spin‐coated, and the PEDOT: BTB film was electrodeposited and then washed off the photoresist with NMP (N‐methy1‐2‐pyrrolidinone) solution. Finally, the photoresist SU‐8 2002 was patterned by developer SU‐8 to passivate the parts other than semiconducting channels and the gate electrode.

### Characterizations of Stacked Layers and OECT‐Based H_2_O_2_ Sensor

Morphological characterizations of PEDOT: PSS and PEDOT: BTB were performed using an atomic force microscope (AFM) and scanning electron microscope (SEM), respectively. The photoelectrochemical properties of stacked PEDOT: BTB/PEDOT: PSS were tested using UV–vis spectroscopy. The CV and electrochemical impedance spectroscopy (EIS) investigations were conducted on an electrochemical workstation. The OECT performance characterizations (including the transfer curve, the transconductance curve, and the transient response) were performed on a source measure unit. The stability and reliability of the proposed sensors were evaluated by measuring the hysteresis width and drift rate. Note that the drift rate investigation followed the hysteresis experiment immediately. The selectivity was then assessed by detecting H_2_O_2_ in other active compounds, such as glucose, lactic acid, and uric acid. These reagents were commonly found in food and biological fluids, possibly interfering with H_2_O_2_ detection.

### Fabrication of OECTs‐Based Ultralow LOD Glucose Sensor

For fabricating the glucose sensor, the gate electrode of OECT was modified with glucose oxidase (GOx), referring to the method described in the literature.^[^ ^16^, ^17^, ^18^ ^]^ In detail, the Pt electrode with a diameter of 2 mm was purchased from Shanghai Chenhua Instruments Co. Ltd. The Pt electrode was patterned using PET tape perforated by a UV laser marking machine (RD‐JW355, Shanghai Cifang Electric Technology Co., Ltd, China). The enzymatic layer was prepared by drop casting 10 µL of a solution containing 20 mg·mL^−1^ of GOx in distilled water onto the commercial Pt electrode of 3.14 mm^2^. Afterward, the enzymatic layer was left to dry for 6 hours at 4 °C. Finally, 10 µL of the Nafion solution was applied to make the upper layer that entrapped the enzymatic layer and let dry overnight at room temperature. The Pt‐Gox‐Nafion stacked electrodes were kept at 4 °C when not in use.

## Conflict of Interest

The authors declare no conflict of interest.

## Supporting information



Supporting Information

## Data Availability

Research data are not shared.
