# Supplementary material for: Ultra‐low LOD H2O2 Sensor Based on Synergistic Nernst Potential Effect
Source: Adv Sci (Weinh). 2025 Apr 17;12(26):2413898. doi: 10.1002/advs.202413898 (PMC12244517; doi:10.1002/advs.202413898)
Supplement: Supplementary file 1 — Supporting Information [file ADVS-12-2413898-s001.docx]

**Ultra-low LOD H_2_O_2_ sensor based on synergistic Nernst potential effect**

**Tables of Contents**

[**Note S1. Reagents, instruments, and characterizations 3**](#_Toc191281353)

[**Note S2. Electrodeposition of PEODT: BTB on PEDOT: PSS film 5**](#_Toc191281354)

[**Note S3. AFM investigations 6**](#_Toc191281355)

[**Note S4 The UV-vis characterizations 7**](#_Toc191281356)

[**Note S5. Comparison of proposed H_2_O_2_ sensor and sensor with state-of-the-art sensors 8**](#_Toc191281357)

[**Note S6. The experimental setup for measuring the electrolyte potential 9**](#_Toc191281358)

[**Note S7. Steady/transient-state response of OECTs and CV investigations 10**](#_Toc191281359)

[**Note S8. The peripheral circuits 12**](#_Toc191281360)

[**Note S9. Traced the H_2_O_2_ level in the actual sample 14**](#_Toc191281361)

[**Note S10. Comparison of proposed glucose sensor with state-of-the-art sensors 16**](#_Toc191281362)

Note S1. Reagents, instruments, and characterizations

PEDOT: PSS, ethylene glycol (EG), dodecyl benzene sulfonic acid (DBSA), 3-glycidoxypropyltrimethoxysilane (GOPS), EDOT, lactic acid, glucose oxidase and contains Nafion^TM^ 1100W perfluorinated resin solution were purchased from Shanghai Aladdin Biochemical Technology Co., Ltd., China. The glucose anhydrous and uric acid were purchased from Shanghai Macklin Biochemical Technology Co., Ltd., China. H_2_O_2_ aqueous solution (approximately 35wt. %) was purchased from Huize Biochemical Co., Ltd., China. The 10 × PBS (Ph=7.4) was purchased from Beijing Lab Lead Biotechnology Co., Ltd., China. BTB, potassium nitrate (KNO_3_), dibasic potassium phosphate (KH_2_PO_4_), potassium hydroxide (KOH), and hydrochloric acid (HCl) were purchased from National Pharmaceutical Group Chemical Reagent Co., Ltd., China. Polyethylene terephthalate (PET) was purchased from Shanghai Kangda Chemical New Material Group Co., Ltd., China. AZ 400K developer, ROL-7133 photoresist, SU-8 2002 developer, SU-8 developer, and NMP solution were purchased from Suzhou Rdmicro Co., Ltd., China.

The starting pH solution was prepared by dissolving 0.4044 g KNO_3_ and 0.544 g KH_2_PO_4_ in 40 ml 0.1 × PBS. Then, the pH level of the aqueous solutions could be tuned between 2 and 9 by adding HCl or KOH. To eliminate the effect of ionic strength on the experimental results, we adjusted the conductivity of all solutions to be σ = 34.39 ± 0.73 mS⋅cm^−1^ assisted with KCl aqueous solution. The pH level and conductivity were confirmed by the SevenExcellence pH/Ion/Cond meter (S475, Mettler Toledo, Switzerland).

To prepare the standard solution of H_2_O_2_, 10 M H_2_O_2_ was diluted with 0.1 × PBS to obtain the desired concentrations, and then we adjusted the conductivity of all solutions to be σ = 34.39 ± 0.73 mS⋅cm^−1^ assisted with KCl aqueous solution. The standard glucose solution was prepared for selectivity investigations by dissolving 9.008 g glucose anhydrose in 50 mL 0.1 × PBS. Then we continued to dilute it to the desired concentrations with 0.1 × PBS adjusting the conductivity to be σ = 34.39 ± 0.73 mS⋅cm^−1^ assisted with KCl aqueous solution; The standard lactic acid solution was prepared by dissolving 0.8406 g lactic acid in 50 ml 0.1 × PBS and diluted to 10^-5^ M with 0.1 × PBS with σ = 34.39 ± 0.73 mS⋅cm^−1^; The standard uric acid solution was prepared by dissolving 0.4504 g uric acid in 50 ml 0.1 × PBS and diluted to 10^-5^ M with σ = 34.39 ± 0.73 mS⋅cm^−1^. It should be noted that the pH values of H_2_O_2_, glucose, lactic acid, and uric acid standard solutions were all adjusted to 7.13 using HCL and KOH.

The device characterizations were performed by the AFM (Bruker Dimension Icon, Bruker, USA), the digital microscope (DSX1000, Olympus, Japan), the SEM (Gemini Sigma 300, Carl Zeiss AG, German), UV-vis (OCEAN-HDX-UV-VIS, Ocean Insight, America), dual constant potential electrochemical workstation potentiostat/galvanostat (VIONIC, Metrohm Autolab, Switzerland).

When investigating the transfer and transconductance curves, the drain voltage (*V*_D_) was set to -0.6 V, the voltage step was 25 mV, each step lasted 0.2 s, and the gate voltage (*V*_G_) was scanned from -0.6 to +0.6 V. When investigating the H_2_O_2_ sensor’s potentiostatic response, *V*_D_ was set at -0.6 V, and *V*_G_ was -0.6 V, +0.6 V, respectively. When conducting the EIS investigations, a sine voltage of 5 mV was applied to the working electrode without DC bias and within the frequency range of 10^0^ - 10^5^ Hz. When investigating the transient responses, *V*_G_ stepped from -0.6 V to + 0.6 V and backed to -0.6 V. When investigating the CV response of various electrodes to H_2_O_2_, the electrode potential was scanned between -0.8 V and +0.8 V, with a scanning speed of 100 mV/s, a sampling interval of 1 mV, and a current sensitivity of 1 mA. When investigating the OCP response, a Pt foil was utilized as the counter electrode, a saturated calomel electrode (SCE) was used as the reference electrode, and the sampling interval was set to 0.2 s.

Note S2. Electrodeposition of PEODT: BTB on PEDOT: PSS film


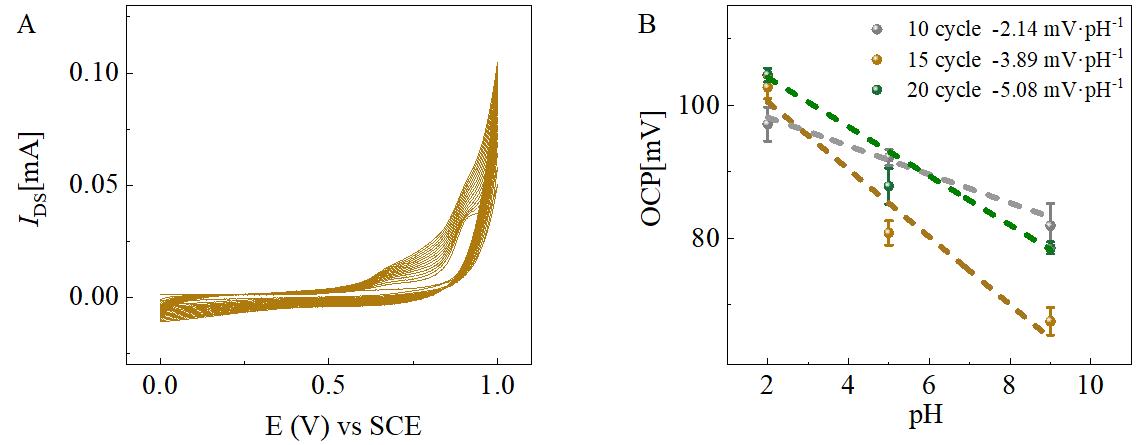


Figure S1. Defined the cycle number for PEODT: BTB electrodeposition. (A) Cyclic voltammograms for the PEDOT: BTB film electrodeposition. (B) The calibrating results of the pH sensitivity of PEDOT: BTB films were obtained when different CV cycles were used for film preparation. Dashed lines were guides to aid visualization. The error bar indicated the standard error of three independent devices. The stacked PEODT: BTB/PEDOT: PSS bilayer prepared by 15 cycles CV electrodeposition presented the highest sensitivity of -5.08 mV·pH^-1^.

Note S3. AFM investigations

**
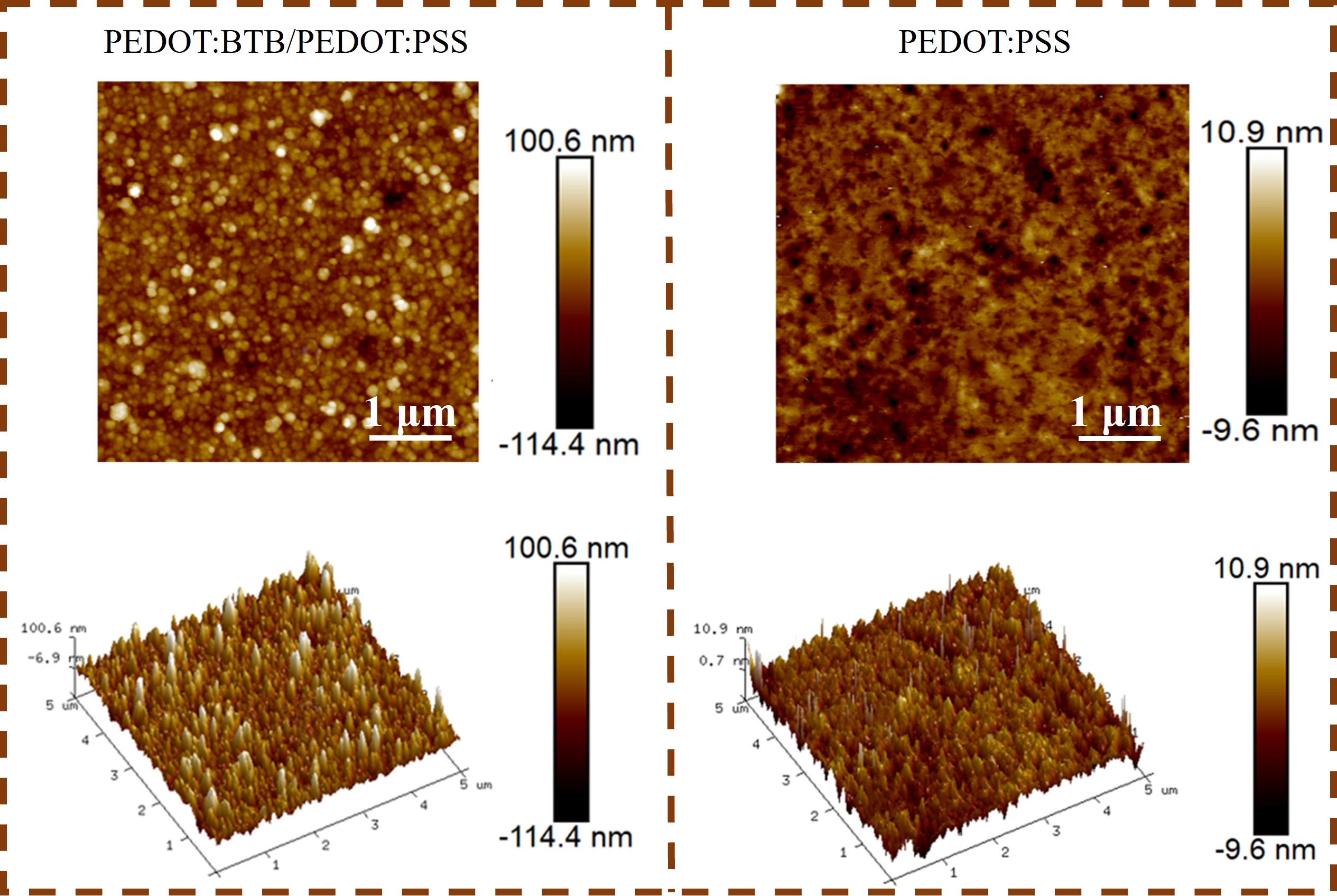
**

Figure S2. The AFM characterizations were performed under the tapping mode. The semiconducting layer’s thickness was evaluated by acquiring topographical images of 5 μm × 5 μm scanning area. The surface roughness was increased from 3.02 nm for the single layer PEDOT: PSS (the right row) to 22.1 nm for the stacked layer of PEDOT: BTB/PEDOT: PSS (the left row).

Note S4 The UV-vis characterizations


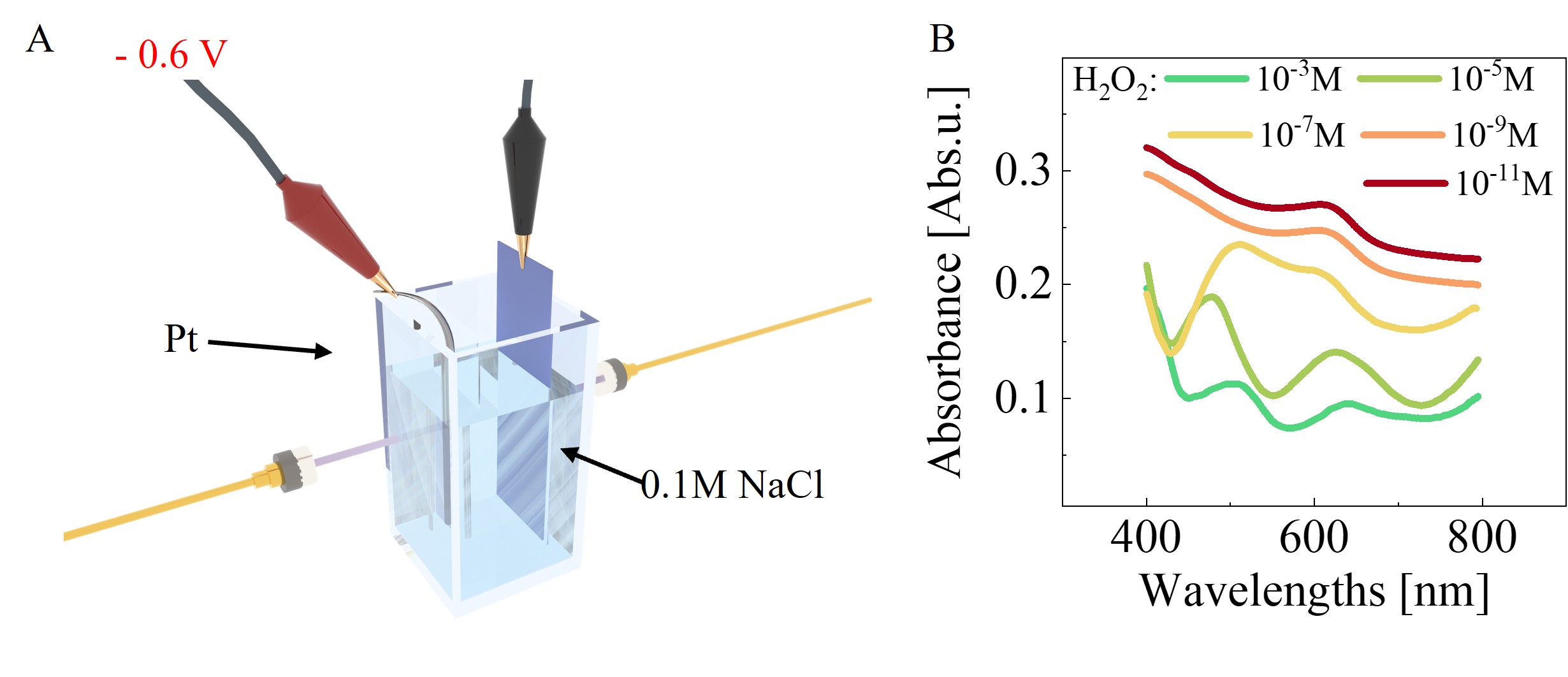


Figure S3. The UV-vis characterizations were conducted by immersing the stacked layer in 0.1 M NaCl mixed with different concentrations of H_2_O_2_ and biasing the Pt electrode with -0.6 V vs the stacked layer.

Note S5. Comparison of proposed H_2_O_2_ sensor and sensor with state-of-the-art sensors

Table S1. Comparison of proposed H_2_O_2_ sensor with state-of-the-art sensors

| Method | | Electrode | | Linear Range | | LOD | | Sensitivity | | Ref |
| --- | --- | --- | --- | --- | --- | --- | --- | --- | --- | --- |
| electrochemistry | | RGO-Pt/GCE | | 5×10^-7^ ~3.475×10^-3^ M | | 2×10^-7^ M | | 459 ± 3 mA·M^−1^·cm^−2^ | | ^[1]^ |
| electrochemistry | | Co3N NW/TM | | 2×10^-6^~2.8×10^-2^ M | | 10^-6^ M | | 139.9 μA·mM^-1^·cm^-2^ | | ^[2]^ |
| electrochemistry | | Pt-ZnO/GCE | | 2×10^-5^~5×10^-3^ M | | 1.5×10^-6^ M | | -3.94 μA·mM^-1^ | | ^[3]^ |
| electrochemistry | | PtPb/G | | 2×10^-9^~2.5×10^-3^ M | | 2×10^-9^ M | | 4.05 mA·mM^-1^·cm^-2^ | | ^[4]^ |
| electrochemistry | | AuNPs/Co-LDH | | 4×10^-6^~1.6×10^-2^ M | | 1.9×10^-7^ M | | 406.61 μA·mM^-1^·cm^-2^ | | ^[5]^ |
| Method | Gate Electrode | | Semiconducting Layer | | Linear Range | | LOD | Sensitivity | Response Time | Ref |
| OECT | Pt NPs/MWCNTs/  CPE | | PEDOT: PSS | | 5×10^-7^~10^-4^ M | | 2.0×10^-7^ M | 0.234 decade^-1^ | 500 s | ^[6]^ |
| OECT | CNT/Pt NPs | | PEDOT: PSS | | 10^-8^~8×10^-7^ M | | / | 6.31×10^-7^ A·dec^-1^ | / | ^[7]^ |
| OECT | Pt-CeO_2_ NS-MWCNT | | PEDOT: PSS | | 10^-7^~10^-4^ M | | 8.28×10^-8^ M | 0.118 decade^-1^ | / | ^[8]^ |
| OECT | Pt | | PEDOT: PSS | | 5 ×10^-6^~10^-3^ M | | 5 μM | / | / | ^[9]^ |
| OECT | Pt | | PEDOT: BTB/  PEDOT: PSS | | 10^-11^~10^-3^ M | | 1.8×10^-12^ M | -1.18×10^-4^ A·dec^-1^ | 46.60 s | This work |

According to the literature,^[1]^ the RGO-Pt/GCE indicated the graphene-Pt nanoparticles/glassy carbon electrode. According to the literature,^[2]^ the Co3N NW/TM indicated the cobalt nitride nanowire /Ti mesh. According to the literature,^[3]^ the Pt-ZnO/GCE indicated the Pt-ZnO/glassy carbon electrode. According to the literature,^[4]^ the PtPb/G indicated the PtPb nanoplates/graphene.

According to the literature,^[5]^ the AuNPs/Co-LDH indicated the gold nanoparticles/cobalt-based layered double hydroxide. According to the literature,^[6]^ the Pt NPs/MWCNTs/CPE indicated the platinum nanoparticles/ carbon nanotubes /screen-printed carbon paste electrode. According to the literature,^[7]^ the CNT/Pt NPs indicated the carbon nanotubes/platinum nanoparticles. According to the literature,^[8]^ the Pt-CeO_2_ NS-MWCNT indicated the platinum-loaded CeO_2_ nanosphere-carbon nanotube-modified gate electrode.

Note S6. The experimental setup for measuring the electrolyte potential


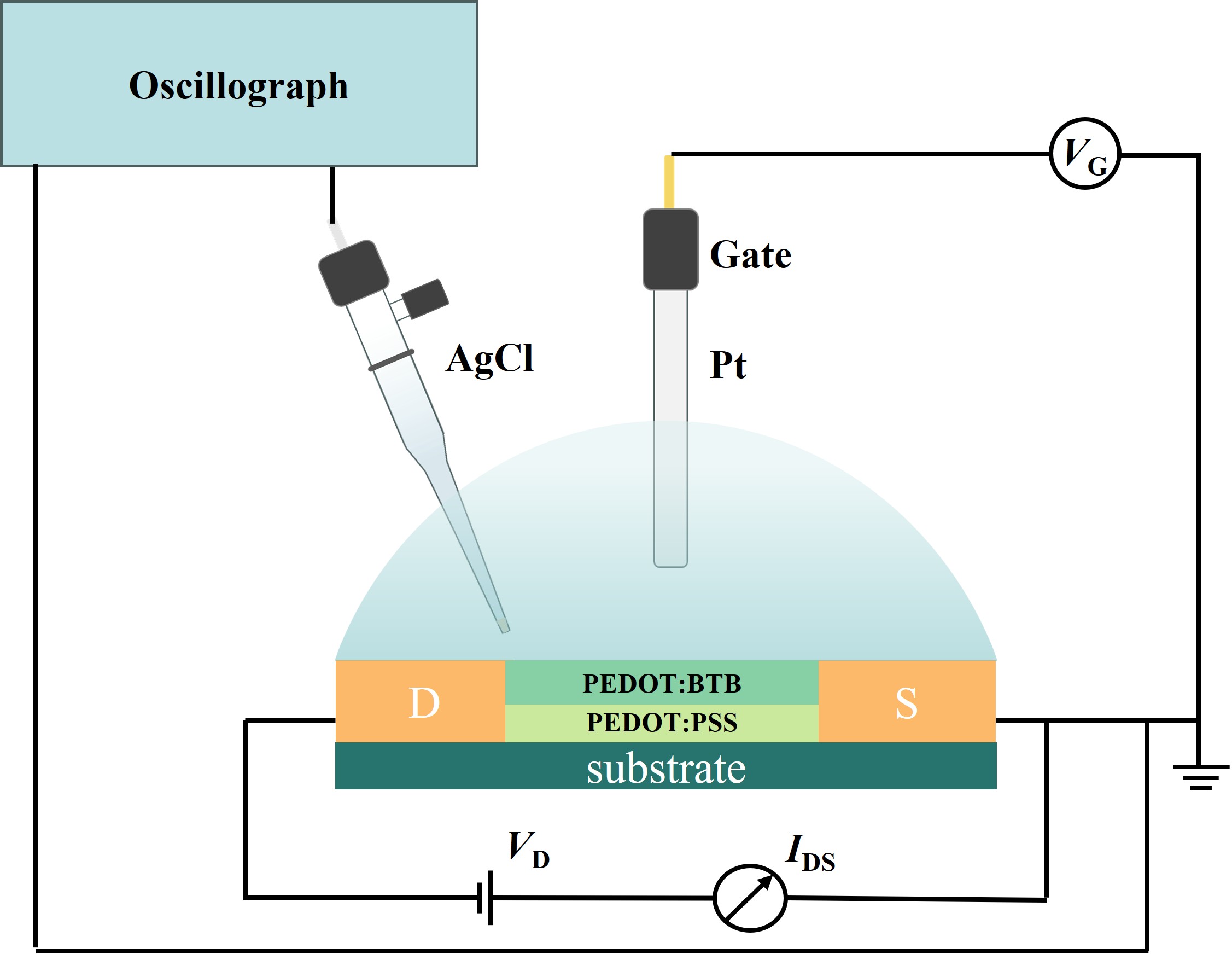


Figure S4. The experimental setup for measuring the electrolyte potential introduced an Ag/AgCl electrode into the electrochemical system.

Note S7. Steady/transient-state response of OECTs and CV investigations


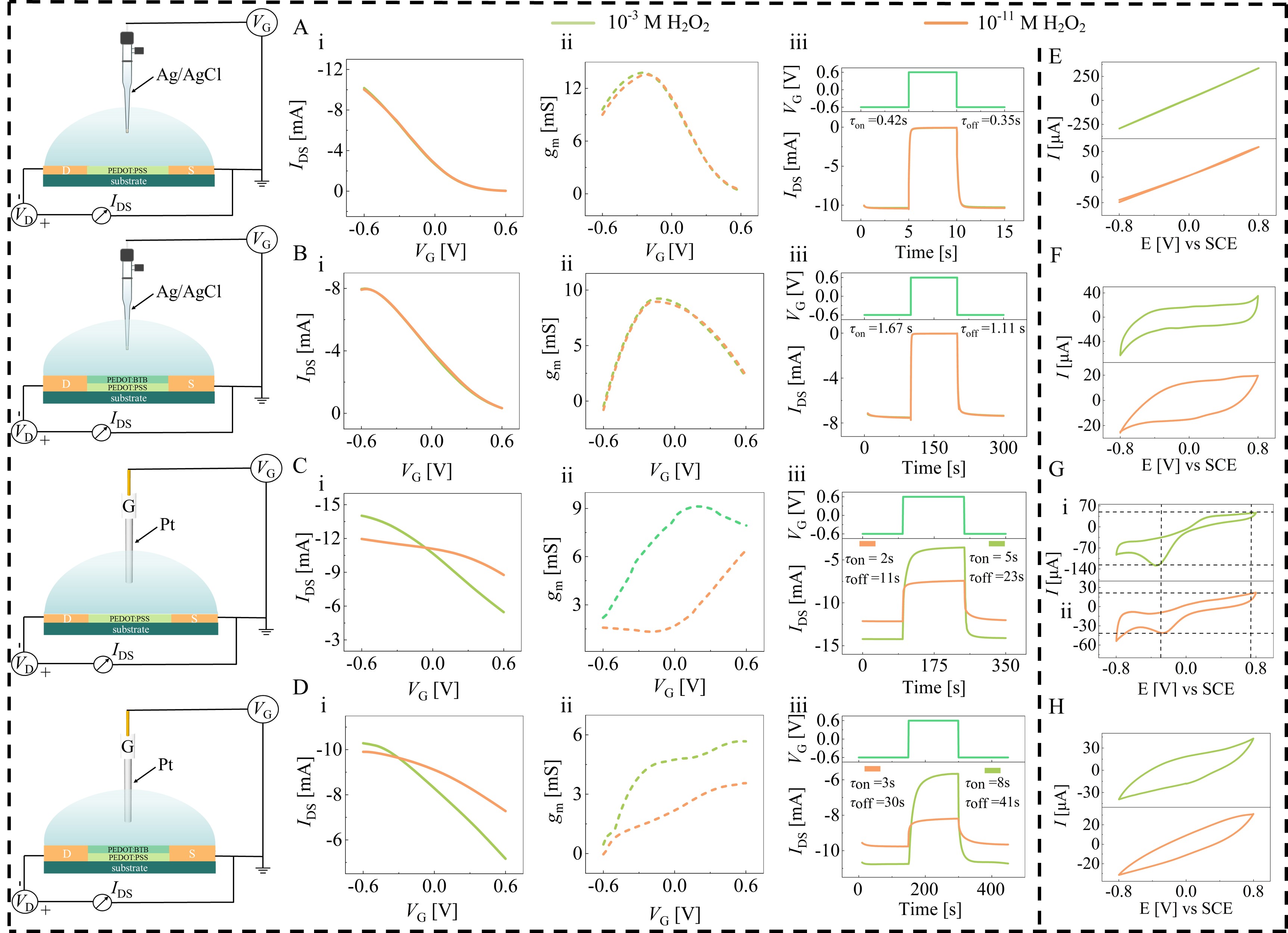


Figure S5. The steady-state and transient-state response of OECTs and CV response of various electrodes exposed to H_2_O_2_. Transfer curves, transconductance curves, and transient responses of OECTs were obtained (A) when the Ag/AgCl electrode was employed as the gate electrode, and the single PEDOT: PSS layer was used as the semiconducting channel; (B) when the Ag/AgCl electrode was employed as the gate electrode, and the stacked PEDOT: BTB/PEDOT: PSS layer was used as the semiconducting channel; (C) when the Pt electrode was employed as the gate electrode and the single PEDOT: PSS layer was used as the semiconducting channel; (D) when the Pt electrode was employed as the gate electrode, and the stacked PEDOT: BTB/PEDOT: PSS layer was used as the semiconducting channel. CV results were obtained when (E) the Ag/AgCl electrode, (F) PEDOT: PSS modified Au electrode, (G) the Pt electrode, or (H) the PEDOT: BTB/PEDOT: PSS modified Au electrode was employed as the working electrode of the three-electrode electrochemical system.

It was worth noting that the traditional Ag/AgCl electrode-gated OECT presented a slower switching-on (+0.6 →-0.6V) than switching-off (-0.6 V→+0.6 V) speed (Figure S5A-ⅲ and S5B-ⅲ).^[10]^ However, this pattern was reversed for the H_2_O_2_ sensor. As shown in Figure S5C-ⅲ and S5D-ⅲ, the switching-off time (*τ*_off_) was more significant than the switching-on time (*τ*_on_), which may be due to the higher catalytic efficiency of Pt electrodes under negative voltage than under positive voltage (Figure 3D). In addition, our previous work found that the time required for the solid-liquid interface between BTB molecules and hydrogen ions to reach equilibrium increased with the decrement of hydrogen ions.^[11]^ During the switching-off dynamic process (-0.6 V→+0.6 V), the semiconducting layer tended to have a negative potential relative to the gate electrode, thus the hydrogen ions were consumed near the semiconducting layer (Equation 4). The dynamic process of H^+^ concentration reduction would further reduce the electrochemical equilibrium rate between H^+^ and BTB molecules, thus resulting in a slow switching-off response speed for the Pt electrode-gated OECTs.

Note S8. The peripheral circuits


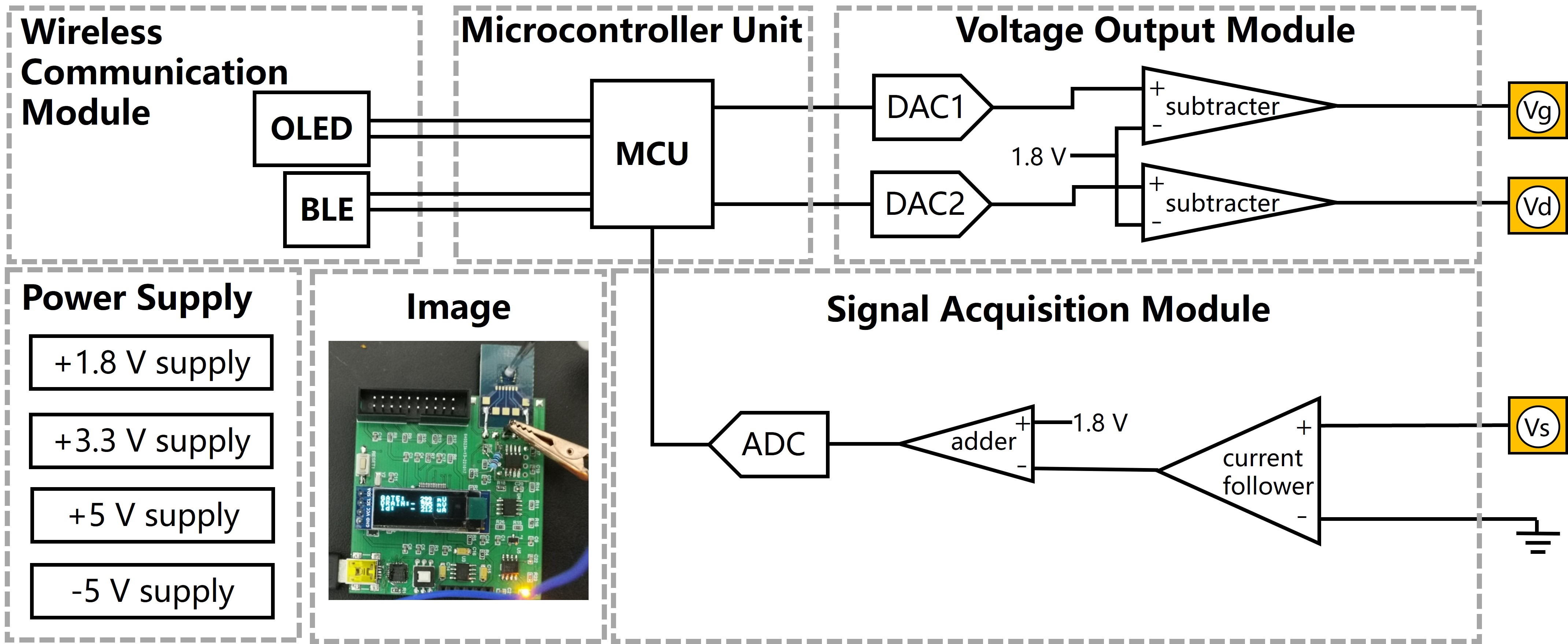


Figure S6. The peripheral circuits.

Five modules were used and integrated into the circuit.

(Ⅰ) Power supply module: The main power supply was +5 V, provided through an external connection via Mini-USB (920-462A2021S10108, Shenzhen Jing Extension of the Electronic Co., ltd). Then, the +5 V voltage was flipped to -5 V by the voltage converter (ICL7660M/TR, HuaGuan Semiconductor Co., ltd). To power the operational amplifier (LM358DR2G, On Semiconductor Co., ltd), both +5 and -5 V voltages were employed. At the same time, the +5 V power supply was then converted to +3.3 V by the voltage regulator (TPS7333QDR, Texas Instruments Co., ltd), which was used to power the microcontroller unit (MCU, STM32F103RCT6, STMicroelectronics Co., ltd)). Then, +3.3 V voltage was converted to +1.8 V by the voltage reference (REF3318AIDBZR, Texas Instruments Co., ltd). Since MCU only outputted analog signals ranging from 0 to +3.3 V, the +1.8 V voltage regulated the input signal of OECT. In addition, the MCU could only handle positive voltage inputs. Thus, the +1.8 V voltage was also used to regulate the analog-to-digital converter’s acquisition signals (ADC).

(Ⅱ) Voltage output module: the voltage output module included two digital-to-analogue converters (DAC) and one subtractor built by an LM358DR2G operational amplifier. The subtractor was employed to control *V*_D_ and *V*_G_, which should be operated in the range of -0.8 V and +0.8 V. High-speed 12-bit DAC produced the output voltage of high resolution and fast scanning speed.

(Ⅲ) Signal acquisition module: the signal acquisition module was a current follower, an analog-to-digital converter, and an adder. The LM358DR2G operational amplifier constructed the current follower and the adder. By combining the current follower with the ADC, the output current of OECT could be collected. The adder added +1.8 V voltage to the acquisition voltage signal, ensuring that the signal input to the MCU was positive.

(Ⅳ) MCU: the STM32F103RCT6 chip was purchased from STMicroelectronics Co., ltd, which was employed to control the working sequence of DAC, ADC, and data transmission. MCU and subtractor together could tune the amplitude of the OECT’s input signal.

(Ⅴ) Extension: A wireless communication module (JDY-08, YouXin Electronic Co., Ltd ) was installed for data exchange and transmission with mobile devices. In addition, a display module (GY091BD01, Shen Zhen Genyu Optical Co., Ltd.) was embedded for real-time data display.

Note S9. Traced the H_2_O_2_ level in the actual sample

We used the commercially available milk as a composite sample to compare the national standard method (GB 5009.226—2016 published by the National Health and Family Planning Commission of China) and our proposed sensor.

The specific comparison procedure was as follows: Firstly, the calibration curves (marked with orange) were obtained by the national standard method and OECT approach, respectively, in the PBS standard solution mixed with H_2_O_2_ of known concentrations. Secondly, the two methods were used to detect the concentration of H_2_O_2_ in the actual sample milk (marked as green dots), and the recovery rates were calculated (Tables S2 and S3).

Before measurement, the sample was sent to a professional testing agency (Xinzhou Comprehensive Inspection and Testing Center) for quantitative analysis using titanium salt colorimetry (GB 5009.226—2016). After confirming that there was no H_2_O_2_ in the original sample, 10 mol/L H_2_O_2_ of different volumes was spiked into the 0.5 mL raw sample, and the mixture was diluted to 50 mL by 0.1 × PBS. Finally, raw samples spiked with 10^-1^,10^-2^, 10^-3^, 10^-4^, 10^-5^, 10^-7^,10^-9^, 10^-11^, 10^-12^ and 10^-13^ M H_2_O_2_ were obtained. As shown in Figure S7, the national standard method could conduct H_2_O_2_ detection within the linear range of 10^-4^ to 10^-2^ M with a LOD of 8.8 × 10^-5^ M.


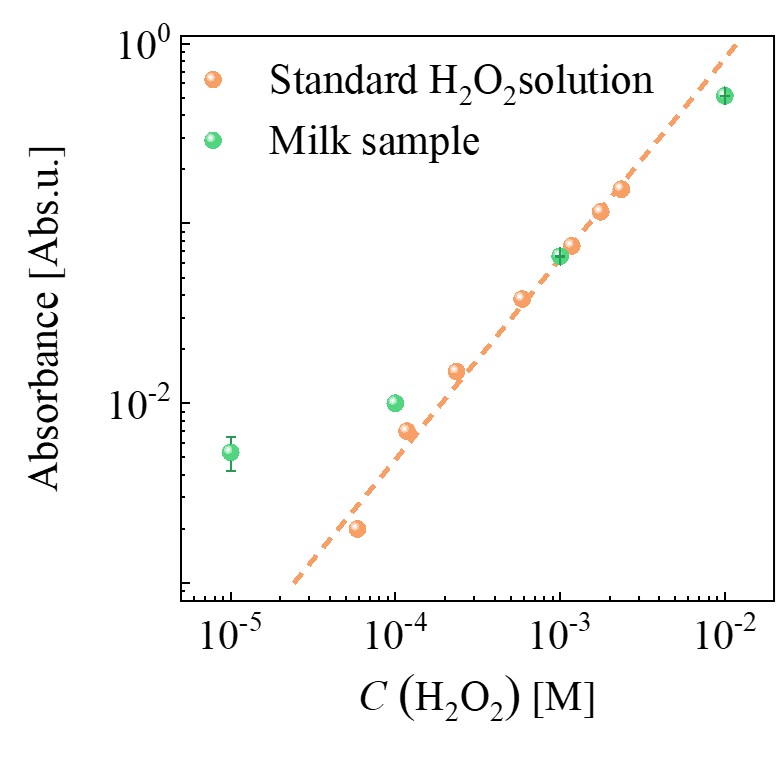


Figure S7. Traced the H_2_O_2_ level in the actual sample by titanium salt colorimetry. The error bar indicated the standard error of three independent devices.

Table S2. The recovery experimental results of the national standard method

| Concentration of H_2_O_2_ in milk samples | Concentration of H_2_O_2_ measured  by the proposed sensors | Recovery (%) | RSD (%) |
| --- | --- | --- | --- |
| 10^-5^ M | 10^-3.99±0.098^ M | 79.93 | 2.46 |
| 10^-4^ M | 10^-3.78^ M | 94.50 | 0 |
| 10^-3^ M | 10^-3.00±0.005^ M | 100.11 | 0.19 |
| 10^-2^ M | 10^-2.12±0.005^ M | 106.16 | 0.27 |

Table S3. The recovery experimental results of our proposed detection method

| Concentration of H_2_O_2_ in milk samples | Concentration of H_2_O_2_ measured  by the proposed sensors | Recovery (%) | RSD (%) |
| --- | --- | --- | --- |
| 10^-11^ M | 10^-10.40±0.349^ M | 94.55 | 3.36 |
| 10^-9^ M | 10^-9.20±0.341^ M | 102.24 | 4.41 |
| 10^-7^ M | 10^-6.67±0.191^ M | 95.34 | 2.87 |
| 10^-5^ M | 10^-4.86±0.300^ M | 97.36 | 6.16 |
| 10^-3^ M | 10^-2.80±0.202^ M | 93.47 | 7.23 |

Note S10. Comparison of proposed glucose sensor with state-of-the-art sensors

Table S4. Comparison of proposed glucose sensor with state-of-the-art sensors

| Method | | Electrode | | Linear Range | | LOD | | Ref | |  |
| --- | --- | --- | --- | --- | --- | --- | --- | --- | --- | --- |
| electrochemistry | | ZnO TPs/MXene | | 5×10^-5^ ~7×10^-4^ M | | 1.7×10^-5^ M | | ^[12]^ | |  |
| electrochemistry | | Ni-Co MOF/Ag/rGO/PU | | 10^-5^~6.6×10^-4^ M | | 3.28×10^-6^ M | | ^[13]^ | |  |
| electrochemistry | | Ag NPs/ZIF-67 @CNT | | 10^-5^~7×10^-3^ M | | 4.6×10^-7^ M | | ^[14]^ | |  |
| electrochemistry | | ZnFe_2_O_4_/NF | | 10^-5^~10^-4^ M | | 8×10^-7^ M | | ^[15]^ | |  |
| electrochemistry | | CuO/M | | 10^-4^~10^-3^ M | | 1.7×10^-8^ M | | ^[16]^ | |  |
| Method | Gate Electrode | | Semiconducting Layer | | Linear Range | | LOD | | Ref | |
| OECT | P-90/GOx | | P-90 | | 10^-8^~2×10^-2^ M | | 10^-8^ M | | ^[17]^ | |
| OECT | PPy/rGO/PA6/GOx/Nafion | | PPy/rGO/PA6 | | 10^-9^~5×10^-6^ M | | / | | ^[18]^ | |
| OECT | CHIT–graphene/GOx/Pt | | PEDOT: PSS | | 10^-8^~10^-3^ M | | 10^-8^ M | | ^[19]^ | |
| OECT | Nafion/GOx/Pt-NPs/TNT | | PEDOT: PSS | | 10^-7^~5×10^-3^ M | | 10^-7^ M | | ^[20]^ | |
| OECT | Pt/PtNPs/GOx:BSA/CHIT | | PrC_60_MA | | 3×10^-5^~10^-2^ M | | 3×10^-5^ M | | ^[21]^ | |
| OECT | Ag/AgCl | | Enzymatic PEDOT-PAH | | 10^-4^~7×10^-3^ M | | 10^-4^ M | | ^[22]^ | |
| OECT | Nafion-Gox-Pt | | PEDOT: BTB/ PEDOT: PSS | | 10^-10^~10^-2^ M | | 8.82×10^-11^ M | | This work | |

According to the literature,^[12]^ the ZnO TPs/Mxene was ZnO tetrapods (ZnO TPs) and Mxene (Ti3C2Tx) nanoflakes mixed preparation. According to the literature,^[13]^ the Ni-Co MOF/Ag/rGO/PU was Ni-Co metal-organic framework/Ag/reduced graphene oxide/polyurethane.

According to the literature,^[14]^ the Ag NPs/ZIF-67 @CNT was prepared by Ag nanoparticles (Ag NPs) photochemical deposited on ZIF-67-decorated carbon nanotube. According to the literature, ^[15]^ the ZnFe_2_O_4_/NF was ZnFe_2_O_4_ spinel magnetic nanoparticles /nickel foam (NF). According to the literature,^[16]^ the CuO/M was CuO/MoS_2_ nanocomposites prepared by a facile solution mixing technique. According to the literature,^[17]^ the P-90 was an NDI-T2 copolymer. According to the literature,^[18]^ the PPy/rGO/PA6 was the PA6 filament modified with polypyrrole nanowires and reduced graphene oxide. According to the literature,^[19]^ the CHIT–graphene/GOx/Pt was prepared by GOx/Pt gate electrodes modified with CHIT acetic acid and graphene aqueous mixed solution.

According to the literature,^[20]^ the Nafion/GOx/Pt-NPs/TNT was TiO_2_ nanotube arrays modified with Pt nanoparticles Pt-NPs and glucose oxidase. According to the literature,^[21]^ the PrC_60_MA was C60,N,N,N-trimethyl-1-(2,3,4-tris(2-(2-methoxyethoxy)ethoxy)phenyl) methanaminium monoadduct. According to the literature,^[22]^ the enzymatic PEDOT-PAH was prepared by the immobilization of glucose oxidase (GOx) onto PEDOT-PAH(polyethylenedioxythiophene-polyallylamine hy­drochloride) via electrostatic interactions.

**Reference**

[1] Y. Zhang, X. Bai, X. Wang, K.-K. Shiu, Y. Zhu, H. Jiang, *Anal. Chem.* **2014**, *86*, 9459.

[2] F. Xie, X. Cao, F. Qu, A. M. Asiri, X. Sun, *Sensors and Actuators B: Chemical* **2018**, *255*, 1254.

[3] X. Ke, G. Zhu, Y. Dai, Y. Shen, J. Yang, J. Liu, *Journal of Electroanalytical Chemistry* **2018**, *817*, 176.

[4] Y. Sun, M. Luo, X. Meng, J. Xiang, L. Wang, Q. Ren, S. Guo, *Analytical chemistry* **2017**, *89*, 3761.

[5] J. Yuan, Q. Chen, Y. Xiao, D. Li, X. Jiang, P. Wu, *Applied Surface Science* **2023**, *630*, 157463.

[6] X. Guo, Q. Cao, Y. Liu, T. He, J. Liu, S. Huang, H. Tang, M. Ma, *Anal. Chem.* **2020**, *92*, 908.

[7] X. Wu, J. Feng, J. Deng, Z. Cui, L. Wang, S. Xie, C. Chen, C. Tang, Z. Han, H. Yu, X. Sun, H. Peng, *Sci. China Chem.* **2020**, *63*, 1281.

[8] J. Liu, T. Kong, Y. Xiao, L. Bai, N. Chen, H. Tang, *Biosensors and Bioelectronics* **2023**, *230*, 115236.

[9] F. Cicoira, M. Sessolo, O. Yaghmazadeh, J. A. Defranco, S. Yang, G. G. Malliaras, *Advanced Materials* **2010**, *22*, 1012.

[10] J. Ji, H. Wang, R. Liu, X. Jiang, Q. Zhang, Y. Peng, S. Sang, Q. Sun, Z. L. Wang, *Nano Energy* **2021**, *87*, 106116.

[11] J. Ji, Z. Wang, F. Zhang, B. Wang, Y. Niu, X. Jiang, Z. Qiao, T. Ren, W. Zhang, S. Sang, Z. Cheng, Q. Sun, *InfoMat* **2023**, *5*, e12478.

[12] V. Myndrul, E. Coy, N. Babayevska, V. Zahorodna, V. Balitskyi, I. Baginskiy, O. Gogotsi, M. Bechelany, M. T. Giardi, I. Iatsunskyi, *Biosensors and Bioelectronics* **2022**, *207*, 114141.

[13] Y. Shu, T. Su, Q. Lu, Z. Shang, Q. Xu, X. Hu, *Anal. Chem.* **2021**, *93*, 16222.

[14] W. Qin, X. Li, Y. Zhang, L. Han, Z. Cheng, Z. Li, Y. Xu, *Journal of Alloys and Compounds* **2022**, *910*, 164878.

[15] C. Fan, L. Chen, R. Jiang, J. Ye, H. Li, Y. Shi, Y. Luo, G. Wang, J. Hou, X. Guo, *ACS Appl. Nano Mater.* **2021**, *4*, 4026.

[16] S. Arunbalaji, R. Vasudevan, M. Arivanandhan, A. Alsalme, A. Alghamdi, R. Jayavel, *Ceramics International* **2020**, *46*, 16879.

[17] D. Ohayon, G. Nikiforidis, A. Savva, A. Giugni, S. Wustoni, T. Palanisamy, X. Chen, I. P. Maria, E. Di Fabrizio, P. M. Costa, *Nature materials* **2020**, *19*, 456.

[18] Y. Wang, X. Qing, Q. Zhou, Y. Zhang, Q. Liu, K. Liu, W. Wang, M. Li, Z. Lu, Y. Chen, D. Wang, *Biosensors and Bioelectronics* **2017**, *95*, 138.

[19] C. Liao, M. Zhang, L. Niu, Z. Zheng, F. Yan, *Journal of Materials Chemistry B* **2013**, *1*, 3820.

[20] J. Liao, S. Lin, Y. Yang, K. Liu, W. Du, *Sensors and Actuators B: Chemical* **2015**, *208*, 457.

[21] C. Shi, X. Jiang, Q. Wang, C. Xiang, X. Dong, L. Chi, L. Huang, *Materials Research Express* **2024**, *11*, 115903.

[22] M. Montero-Jimenez, J. R. N. Recky, C. von Bilderling, J. Scotto, O. Azzaroni, W. A. Marmisollé, *Journal of Electroanalytical Chemistry* **2025**, *978*, 118867.
